# Early Administration of Methylene Blue Attenuates Mitochondrial Dysfunction and Cognitive Deficits in a Model of Huntington’s Disease

**DOI:** 10.3390/biology15201801

**Published:** 2026-10-09

**Authors:** Maribel Huerta-Cervantes, Donovan J. Peña-Montes, Miguel Ángel López-Vázquez, Alfredo Saavedra-Molina, María Isabel Pérez-Vega, María Esther Olvera-Cortés

**Affiliations:** 1 Laboratorio de Psicobiología, Departamento de Ciencias de la Tierra y de la Vida, Centro Universitario de los Lagos, Universidad de Guadalajara, Lagos de Moreno 47000, Jalisco, Mexico; marzy112@yahoo.com.mx; 2 Laboratorio de Neurofisiología Clínica y Experimental, Centro de Investigación Biomédica de Michoacán, Instituto Mexicano del Seguro Social, Morelia 58341, Michoacán, Mexico; 3 Laboratorio de Neurofisiología del Músculo Esquelético, Centro Universitario de Investigaciones Biomédicas, Universidad de Colima, Colima 28040, Colima, Mexico; dpena11@ucol.mx; 4 Laboratorio de Neuroplasticidad, Centro de Investigación Biomédica de Michoacán, Instituto Mexicano del Seguro Social, Morelia 58341, Michoacán, Mexico; migangelv@yahoo.com.mx; 5 Laboratorio de Bioquímica, Instituto de Investigaciones Químico-Biológicas, Universidad Michoacana de San Nicolás de Hidalgo, Morelia 58030, Michoacán, Mexico; francisco.saavedra@umich.mx

**Keywords:** Huntington’s disease, methylene blue, 3-nitropropionic acid, cognitive performance, striatum, mitochondrial function, neuroprotection

## Abstract

Huntington’s disease (HD) is a fatal neurodegenerative disorder marked by progressive motor, cognitive, and psychiatric impairments, for which no disease-modifying therapies exist. Mitochondrial dysfunction is an early feature of HD, highlighting the need to target bioenergetic deficits early. We evaluated the neuroprotective effects of methylene blue (MB) in a 3-nitropropionic acid (3-NP)-induced HD-like rat model characterized by mitochondrial dysfunction and striatal injury. Male Wistar rats received MB for 14 days during 3-NP administration. Behavioral performance was assessed, followed by biochemical and histological analyses of the striatum. MB improved cognitive performance and partially improved specific aspects of motor coordination. At the mitochondrial level, MB restored Complex II activity, membrane potential index, reduced reactive oxygen species (ROS), and modulated mitochondrial function, but did not rescue Complex I. Histologically, MB attenuated striatal histopathological damage. These results suggest that methylene blue has neuroprotective effects in the early stages, particularly at the mitochondrial and cognitive levels. Its limited impact on other behaviors and systemic parameters could indicate that its efficacy depends on timing or duration. Overall, this study supports early mitochondrial targeting in HD therapy and shows methylene blue as a promising, though partial, disease-modifying candidate.

## Simple Summary

Huntington’s disease is an inherited disorder that gradually damages the brain, causing problems with movement, memory, and behavior. At present, no approved treatment has been shown to halt or slow disease progression. Because damage to cellular energy-producing structures occurs very early, treating these changes before severe symptoms appear may help protect the brain. In this study, we evaluated whether methylene blue, a compound known to support energy production and reduce harmful oxidative damage, could protect the brain in an experimental model that reproduces the early stage of Huntington’s disease. Rats treated with methylene blue showed improved memory, neuronal protection and healthier mitochondrial function, together with reduced oxidative damage and partial preservation of brain tissue. However, the treatment had limited effects on motor performance. These findings suggest that methylene blue may help delay some of the earliest changes associated with Huntington’s disease, particularly those affecting brain energy metabolism and cognition. This work supports further research into early treatments aimed at slowing disease progression and improving the quality of life of affected individuals.

## 1. Introduction

Huntington’s disease (HD) is a rare, progressive, autosomal dominant, and ultimately fatal neurodegenerative disorder caused by an expanded CAG trinucleotide repeat in the HTT gene, which results in the production of a mutant huntingtin protein that primarily affects the striatum and leads to characteristic motor, cognitive, and psychiatric manifestations that severely impact quality of life [1,2]. In Mexico, epidemiological data on Huntington’s disease (HD) remain limited, and no national registry is currently available. A prevalence of approximately 4 cases per 100,000 individuals has been reported in Mexico City; however, this estimate cannot be generalized to the national level [3]. Globally, HD prevalence has been estimated at approximately 4.9 cases per 100,000 individuals (95% CI: 3.38–7.06), although substantial heterogeneity exists across populations [4].

Multiple mechanisms contribute to disease progression; converging evidence highlights early mitochondrial dysfunction and redox imbalance as central components of HD pathophysiology [5,6]. Striatal medium spiny neurons exhibit high metabolic demand and limited antioxidant capacity, resulting in increased vulnerability to energetic failure and oxidative stress [5,7]. Mutant huntingtin impairs mitochondrial respiration, disrupts electron transport chain (ETC) efficiency, alters mitochondrial dynamics, and suppresses mitochondrial biogenesis pathways, including PGC-1α signaling [8,9].

Consistent with these alterations, studies in striatal and cortical samples have reported reduced activity of mitochondrial respiratory chain enzymes, particularly complexes II and III [10,11]. These deficits lead to reduced ATP availability and increased generation of reactive oxygen species (ROS), disruption of redox signaling, and oxidative injuries to lipids, proteins, and mitochondrial DNA has been documented in HD postmortem tissue, supporting the role of oxidative stress as a driving event in the disease [5,12,13,14,15]. Notably, mitochondrial and redox disturbances emerge during the early phase, prior to significant neuronal loss, indicating a critical window for early therapeutic intervention. In this context, the mitochondrial toxin 3-nitropropionic acid (3-NP), an irreversible inhibitor of succinate dehydrogenase (complex II), reproduces a biochemical defect characteristic of HD [16]. When administered under subchronic, low-dose regimens, 3-NP induces selective striatal mitochondrial dysfunction, increases oxidative stress, and produces behavioral impairments, including motor alterations and cognitive deficits, without causing extensive cell death [16,17]. Together, these mitochondrial, oxidative, and behavioral alterations make this model a robust mimic of early HD and a suitable platform for evaluating interventions targeting mitochondrial and redox dysfunction.

Methylene blue (MB) has emerged as a promising mitochondria-targeted redox modulator. Through reversible redox cycling, MB can act as an alternative electron carrier, bypassing impaired ETC components, reducing electron leakage, limiting ROS generation, and supporting ATP synthesis. In addition, MB readily crosses the blood–brain barrier and accumulates in mitochondria, where it enhances electron transport efficiency and attenuates oxidative stress [18,19,20,21]. MB and its derivatives have been evaluated clinically, primarily for Alzheimer’s disease and behavioral-variant frontotemporal dementia. Phase II and III trials of compounds including LMTM/HMTM (leuco methylthioninium hydra methanesulfonate/hydromethylthionine mesylate) produced mixed results: several randomized studies failed to meet prespecified cognitive or functional endpoints, although subgroup, exposure-response, and biomarker analyses indicated possible biological or clinical effects in selected populations [22,23,24,25]. These studies mainly targeted pathological protein aggregation, whereas the present study examines MB as a mitochondrial redox modulator.

Evaluation of MB in a model of HD allows direct assessment of how early mitochondrial modulation affects functional and structural outcomes. This study evaluated the effects of MB on striatal mitochondrial function, reactive oxygen species (ROS) production, histopathological alterations, and behavioral performance, including motor function, anxiety- and depression-like behaviors, and short-term memory, in a 3-NP-induced rat model of early-HD (HD-like).

## 2. Materials and Methods

### 2.1. Animals

Fifty-six Wistar adult male rats (16 weeks old) were obtained from the institutional breeding facility and housed under standard conditions (temperature 22 ± 2 °C, relative humidity 55 ± 5%, and a 12 h light/dark cycle) with free access to food and water. Animals were acclimatized to handling for two weeks before experimental procedures began. The experimental protocol was approved by the Ethics and Research Committee of the Instituto Mexicano del Seguro Social with folio R-2021-1602-035 and performed in accordance with the “Norma Oficial Mexicana para el uso de animales de laboratorio” (NOM-062-ZOO-1999) and, with the National Institutes of Health Guide Regarding the Care and Use of Laboratory Animals (NIH Publication no. 80-23). Every possible measure was taken to minimize the animals’ suffering.

HD model was induced by systemic administration of 3-nitropropionic acid (3-NP; Sigma-Aldrich, St. Louis, MO, USA), a mitochondrial toxin that reproduces key biochemical and neuropathological features of Huntington’s disease. Rats receiving 3-NP are hereafter referred to as the HD-like group. Animals in the HD-like groups received intraperitoneal injections of 3-NP at 10 mg/kg for 14 consecutive days. Methylene blue (MB; Sigma-Aldrich, St. Louis, MO, USA) was administered orally via an oral cannula as an aqueous solution at 4 mg/kg/day dose for 14 consecutive days, starting 1 h after the 3-NP injection and continuing throughout the experimental period. The MB dose and route of administration were selected based on previous studies demonstrating its neuroprotective efficacy in neurodegenerative experimental models [26,27,28].

The control group received intraperitoneal saline injections and oral administration of water, while the MB group received oral methylene blue and intraperitoneal saline. The HD-like + MB group received both 3-nitropropionic acid plus methylene blue. Rats were randomly assigned to four experimental groups: control (*n* = 14), HD-like (3-NP, *n* = 13), HD-like + MB (3-NP plus methylene blue, *n* = 13), and MB (methylene blue, *n* = 16). The sample size (*n*) reported throughout the manuscript corresponds to the final number of animals that survived until the end of the experimental protocol and were included in the behavioral, biochemical, and histological analyses. We performed behavioral evaluations in all animals. Subsequently, each group was randomly divided into two subgroups: (1) designated for biochemical assays and (2) designated for histological analyses.

### 2.2. Isolation of Striatal Mitochondria

The bilateral striatum from freshly collected brains was rapidly dissected. Next, homogenized at 4 °C using a Potter–Elvehjem-type glass homogenizer in isolation buffer [220 mM mannitol, 70 mM sucrose, 2 mM 3-(N-morpholino) propanesulfonic acid (MOPS), 1 mM ethyleneglycol-bis (2-aminoethylether)-N,N,N′,N′-tetraacetic acid (EGTA), and 0.25% *w*/*v* bovine serum albumin (BSA), pH 7.4].

Mitochondrial fractions were isolated by differential centrifugation, following the method of Saavedra-Molina and Devlin [29] with minor modifications. The homogenate was centrifuged at 500× *g* for 10 min to remove cellular debris. The resultant supernatant was subsequently centrifuged at 12,000× *g* for 10 min to yield the mitochondrial fraction. The final pellet was gently resuspended in an isotonic mitochondrial buffer (220 mM mannitol, 70 mM sucrose, 2 mM MOPS, pH 7.4). Protein concentration was measured by a modified Biuret assay [30] with BSA as the standard. Mitochondria were used immediately for functional analysis or stored at −70 °C.

### 2.3. Mitochondrial Membrane Potential Assessment

The mitochondrial membrane potential (ΔΨm) was determined using the fluorescent probe safranin according to Figueira et al. [31]. Freshly isolated mitochondria (300 µg protein) were resuspended in assay buffer containing 250 mM sucrose, 10 mM HEPES, 200 µM EGTA, 2 mM KH_2_PO_4_, and 1.4 mM MgCl_2_ (pH 7.4). Safranin (5 µM) was added to the suspension. Baseline fluorescence was recorded at excitation/emission wavelengths of 495/580 nm, respectively, at 25 °C. Subsequently, 5 mM succinate was added as an FADH_2_-linked respiratory substrate, and fluorescence quenching was monitored until a steady-state signal was achieved. Finally, 1 µM carbonyl cyanide m-chloro-phenyl hydrazone (CCCP) was added to dissipate ΔΨm and confirm membrane depolarization. A mitochondrial membrane potential index (Q) was calculated as the fluorescence increase following CCCP addition relative to the post-CCCP fluorescence and expressed as a percentage, using mean fluorescence values before and after uncoupling.

### 2.4. Determination of Mitochondrial Complex I and II Enzymatic Activities

The enzymatic activities of mitochondrial complexes were determined according to the methods described by Peña-Montes et al. using mitochondria previously stored at −70 °C [32]. Prior to the assessment of complexes, I and II, mitochondria were subjected to three freeze–thaw cycles followed by osmotic shock to induce membrane permeabilization. Briefly, 200 µg of mitochondrial protein was resuspended and incubated for 7 min in 250 mM potassium phosphate buffer (KPB, pH 7.5) containing 100 µg bovine serum albumin (BSA), 2.5 µM antimycin A, and 500 µM potassium cyanide (KCN). Freshly prepared 10 mM potassium ferricyanide (K_3_[Fe(CN)_6_]) was then added. The reaction was initiated by adding 100 µM NADH. The decrease in NADH fluorescence was recorded spectrofluorometrically at excitation/emission wavelengths of 352/464 nm, using a Shimadzu RF-5301PC spectrofluorometer (Shimadzu Corporation, Kyoto, Japan). Complex I activity was expressed as rotenone-sensitive activity and was calculated by subtracting the enzymatic activity measured in the presence of 10 µM rotenone from the total activity measured in independent assays.

Mitochondrial complex I activity was expressed as µmol NADH oxidized per minute per mg of protein. Mitochondrial complex II activity was expressed as µmol 2,6-dichlorophenolindophenol (DCIP) reduced per minute per mg of protein. Briefly, 200 µg of mitochondrial protein was incubated for 5 min in 250 mM potassium phosphate buffer containing 200 µg bovine serum albumin (BSA), 5 mM succinate, 5 µM rotenone, 2.5 µM antimycin A, and 0.5 mM potassium cyanide (KCN). The reaction was initiated by the addition of 80 µM DCIP. Enzymatic activity was monitored spectrophotometrically at 600 nm using a PerkinElmer UV/Vis Lambda 18 spectrophotometer (PerkinElmer Inc., Waltham, MA, USA). Independent assays were performed using specific inhibitors to estimate the enzymatic activity of each respiratory chain complex. Rotenone (10 µM) was used for complex I inhibition, 2-thenoyltrifluoroacetone (500 µM) for complex II inhibition, antimycin A (2.5 µM) All assays were performed at 30 °C.

### 2.5. Mitochondrial ROS Production

Reactive oxygen species (ROS) generation was determined according to Huerta-Cervantes et al. [33], using the cell-permeable fluorescent probe 2′,7′-dichlorodihydrofluorescein (H_2_DCFDA) in the presence of an FADH_2_-linked respiratory substrate. Fresh mitochondria (150 µg/mL of mitochondrial protein) were resuspended in assay buffer containing 10 mM HEPES, 100 mM KCl, 3 mM MgCl_2_, and 3 mM KH_2_PO_4_ (pH 7.4). H_2_DCFDA (12.5 µM) was added, and samples were incubated for 10 min on ice under constant agitation. Basal fluorescence was recorded for 1 min, after which 5 mM succinate was added as a respiratory substrate, and fluorescence was monitored for 10 min. Subsequently, antimycin A was added, and fluorescence was recorded for an additional 10 min. Measurements were performed at excitation/emission wavelengths of 480/520 nm using a Shimadzu RF-5301PC spectrophotometer. Activity was calculated following the addition of succinate for 10 min, and the baseline fluorescence recorded before the addition of the FADH_2_-linked substrate, succinate, was subtracted. Results were expressed as arbitrary units per mg of protein.

### 2.6. Histological Analysis

Rats were intracardially perfused with a phosphate buffer containing procaine (1 g/L) and heparin (1000 U/L), followed by a fixative of 4% paraformaldehyde in phosphate buffer (30 mM Na_2_HPO_4_, 30 mM NaH_2_PO_4_, 390 mM NaCl, pH 7.2). Brains were fixed in 4% paraformaldehyde, dehydrated, cleared, and embedded in paraffin blocks. Coronal sections (10 µm thick) were obtained with a microtome. Sections were mounted on glass slides, deparaffinized, rehydrated, and stained with cresyl violet to assess cellular density in predefined striatal regions. The stereotaxic coordinates analyzed were: Coordinate 1 (+1.70 mm from bregma), Coordinate 2 (+1.20 mm from bregma), and Coordinate 3 (+0.70 mm from bregma). Images were acquired under standardized conditions using a Leica light microscope equipped with a digital camera. Cellular density was quantified at 40× magnification by analyzing five non-overlapping fields per section and 5 sections per animal. Data were expressed as cellular density (number of cells per 10,000 µm^2^).

### 2.7. Behavioral Methods

**Figure 1:**
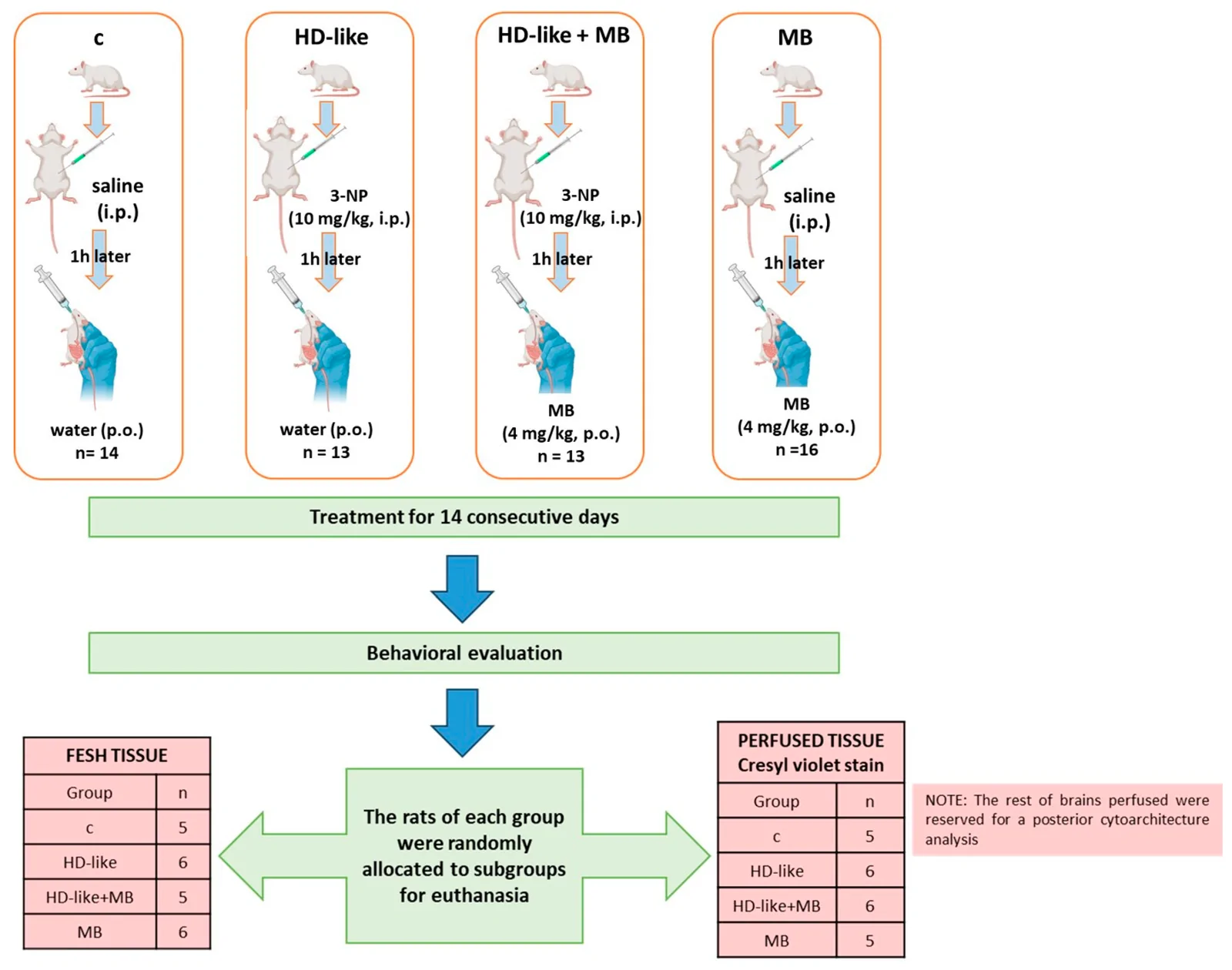
Schematic illustration of the experimental study design in rats.

**Figure 2:**
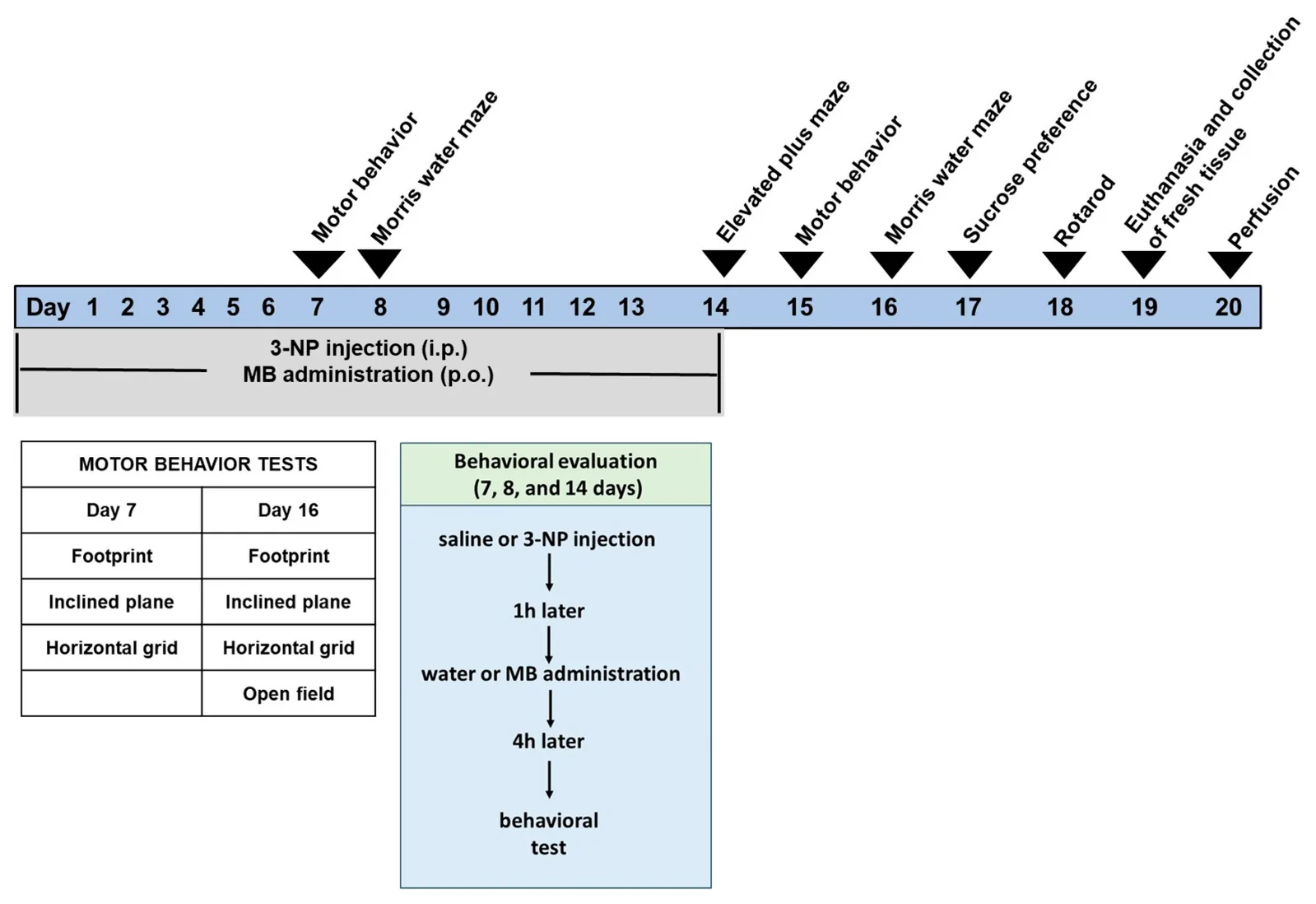
Experimental timeline; i.p., intraperitoneal; p.o., oral administration.

A comprehensive battery of behavioral assessments was conducted to evaluate motor function, anxiety-like behavior, anhedonia, and spatial working memory. Assessments were performed at two stages: a mid-treatment evaluation on days 7 and 8 to detect early functional changes, and a final evaluation conducted from days 14 to 18. On days 7, 8, and 14, behavioral testing was performed 4 h after MB administration. From day 15 onward, after completion of the treatment protocol, behavioral tests were conducted between 08:00 and 16:00 h. Rats were habituated to the testing room for 30 min before each session, and all tests were video-recorded for subsequent analysis (Figure 1 and Figure 2).

#### 2.7.1. Motor Coordination, Balance, and Muscular Strength

A footprint test was employed to evaluate gait patterns. The hind and forepaws were coated with a non-toxic dye, and the rat was then allowed to walk along a narrow paper corridor (15 × 20 cm wide/high and 60 cm long). Three stride lengths of consecutive sets were measured; gait abnormalities were interpreted as indicators of motor impairment.

Motor coordination and balance were assessed using a rotarod operating at fixed rotational speeds. Each rat completed three trials at each rpm (30, 35, 40, and 45 rpm). Rats were placed on the rotating rod and remained until they fell or reached the maximum trial duration of 180 s. A five-minute rest interval was provided between trials to prevent fatigue. Latency to fall was averaged across trials for each rpm.

Postural stability and righting ability were assessed using the inclined plane test with an adjustable base for each tilt angle (30, 35, 40, and 45 degrees). For each inclination, the rats were positioned in three initial orientations: (1) head down, (2) facing left, and (3) facing right, to evaluate directional differences. All rats underwent three trials for each tilt angle and orientation. We recorded two parameters: the latency to turn, the time the rat needed to reorient itself to face upward, and the latency to climb, the time required for the rat to initiate and sustain upward movement, ultimately reaching the platform’s upper area. Trials were terminated after a maximum of 30 s or if the animal slipped from the surface.

Grip strength and fine motor coordination were evaluated using a horizontal wire grid (25 × 35 cm) elevated 50 cm above a padded surface. Each rat was allowed to grasp the grid freely. The time until the rat fell was recorded, with a maximum of 30 s to prevent fatigue. Three trials were conducted per animal, and the mean latency was used for analysis. A shorter latency indicates reduced grip strength or impaired coordination.

Spontaneous locomotor activity was assessed using the open field test. The apparatus consisted of a square arena (60 × 60 cm) divided into eight peripheral quadrants (including the corner areas) and a central area. A 60-W white lamp was positioned above the arena center. Each rat was individually placed in the center of the arena and allowed to freely explore for 10 min. Animal behavior was recorded and analyzed using SciWorks V9.0 software (DataWave Technologies Inc., Longmont, CO, USA). The total distance traveled and time spent in the central and peripheral areas were recorded.

#### 2.7.2. Anxiety-like Behavior, Anhedonia, and Spatial Working Memory

Anxiety-like behavior was assessed using the elevated plus maze, consisting of two closed arms (50 × 10 × 40 cm) and two open arms (50 × 10), elevated 50 cm above the floor. The central area was illuminated with approximately 500 lux. Each rat was gently placed in the central area facing an open arm and allowed to explore for 10 min. The behavior was recorded using Sci-Works software (DataWave Technologies Inc., Longmont, CO, USA). The time spent in closed arms was used as an anxiogenic behavior.

Anhedonia-like behavior was evaluated using the sucrose preference test under conditions designed to minimize physiological stress. Animals were individually housed and simultaneously provided with two bottles, one containing tap water and the other a 1% (*w*/*v*) sucrose solution, for 24 h with ad libitum access to food and water. No prior habituation to the sucrose solution was performed, as the objective was to assess spontaneous sucrose preference under identical experimental conditions for all groups. To minimize positional bias, the location of the sucrose and water bottles was alternated for each animal throughout the test. At the end of the testing period, fluid consumption was determined, and sucrose preference was calculated as the percentage of sucrose solution intake relative to the total fluid intake.

**Figure 3:**
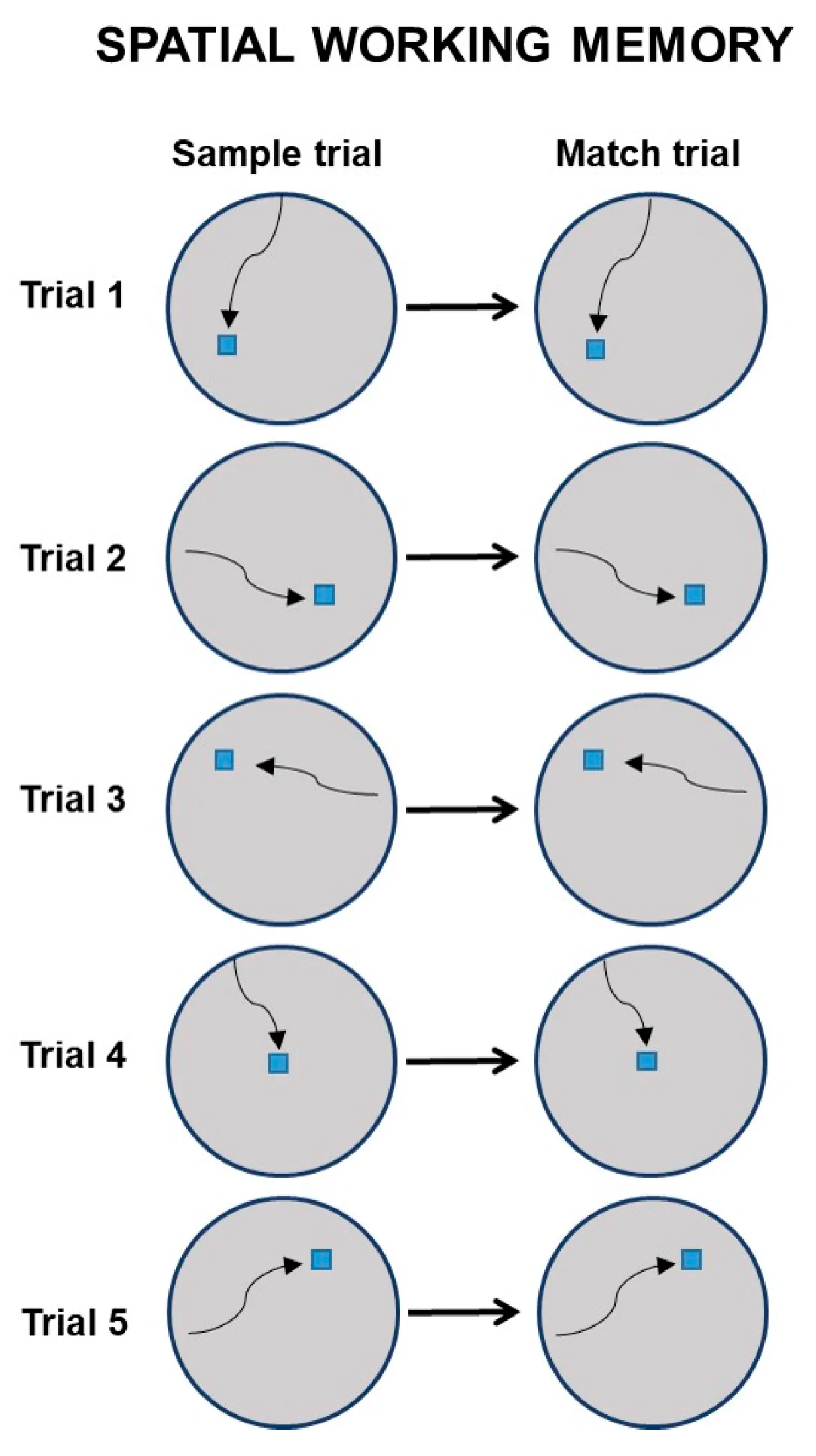
Morris water maze design for the spatial working memory test.

Spatial working memory was evaluated using a modified version of the Morris water maze, in which the platform’s location varied across trials (Figure 3). The maze was a circular pool measuring 150 cm in diameter and 50 cm in height. It was filled with blue-dyed water (gentian violet) and maintained at 27° ± 2 °C. Each rat completed five sets, each consisting of a sample trial followed by a match trial (match-to-sample phase). The platform’s location was unique for each trial pair. Each set began from a distinct starting position within the maze (Figure 2). During the sample trial, rats had 60 s to find and climb the hidden platform. After finding it, they remained there for 15 s. If a rat failed, it was gently guided to the platform and held there for 15 s to acquire spatial cues. A 20-s inter-trial interval, during which the animal rested in a holding cage, separated each sample and match trial. The match trial used the same platform location as the preceding sample trial. A 2-min rest interval was given between each pair of sample/match trials. Swim path lengths were determined using Sci-Works V9.0 software (DataWave Technologies Inc., Longmont, CO, USA). Escape latency and distance traveled were quantified to assess the efficiency of spatial working memory.

### 2.8. Statistical Analysis

Inclined plane, horizontal grid, and rotarod data were normalized using the natural logarithm prior to analysis. Differences among groups in motor performance, anxiety-like behavior, sucrose preference, biochemical determinations, and nuclear density were analyzed using one-way analysis of variance (ANOVA) followed by Tukey’s post hoc test when appropriate. Escape latencies and rotarod data were compared between groups using the Kruskal–Wallis test followed by the Mann–Whitney U test, whereas intragroup comparisons were performed using the Wilcoxon signed-rank test. For the average total distance, statistical comparisons were performed using two-way ANOVA for repeated measures, followed by Bonferroni’s multiple comparisons test. Body weight was analyzed using two-way repeated-measures ANOVA followed by Tukey’s post hoc test. Statistical analyses were performed using GraphPad Prism version 7.0 (GraphPad Software Inc., San Diego, CA, USA). A *p*-value < 0.05 was considered statistically significant. Behavioral data from days 7, 8 and 14–18 were analyzed separately, as each day was considered a predefined evaluation point. The objective was to assess treatment effects at each time point rather than longitudinal changes between days.

## 3. Results

During the experimental period, mortality occurred exclusively in the 3-NP-treated groups. In the HD-like group, 26 rats were initially included; 12 died during the experimental period, leaving 14 animals for subsequent analyses, corresponding to a mortality rate of 46.15%. In the HD-like + methylene blue group, 23 rats were initially included; 8 died, leaving 15 animals, corresponding to a mortality rate of 34.78%. No mortality was observed in the control or MB groups. One rat from the HD-like group and two from the HD-like + MB group were excluded from the study because they did not display the expected behavioral response during testing. These animals showed no apparent mobility impairment during routine observation in their home cages. The sample size (*n*) reported in the subsequent analyses corresponds to the final number of animals that survived until the end of the experimental protocol and were included in the behavioral, biochemical, and histological analyses.

### 3.1. Body Weight Is Reduced in the HD-like Group and Is Not Modified by Methylene Blue

Body weight was monitored throughout the experimental period (Figure S1). The HD-like group showed a progressive reduction in body weight relative to control, reaching significance on day 11 (*p* = 0.04) and persisting through day 13 (*p* = 0.02). MB treatment did not alter this trajectory in HD-like animals, as no differences were observed between the HD-like and HD-like + MB groups. Consistent with this, the HD-like + MB group also had lower body weight than control over the same period in days 11 and 13 (*p* = 0.004 and 0.0007).

### 3.2. Mitochondrial Bioenergetic and Redox Function

**Figure 4:**
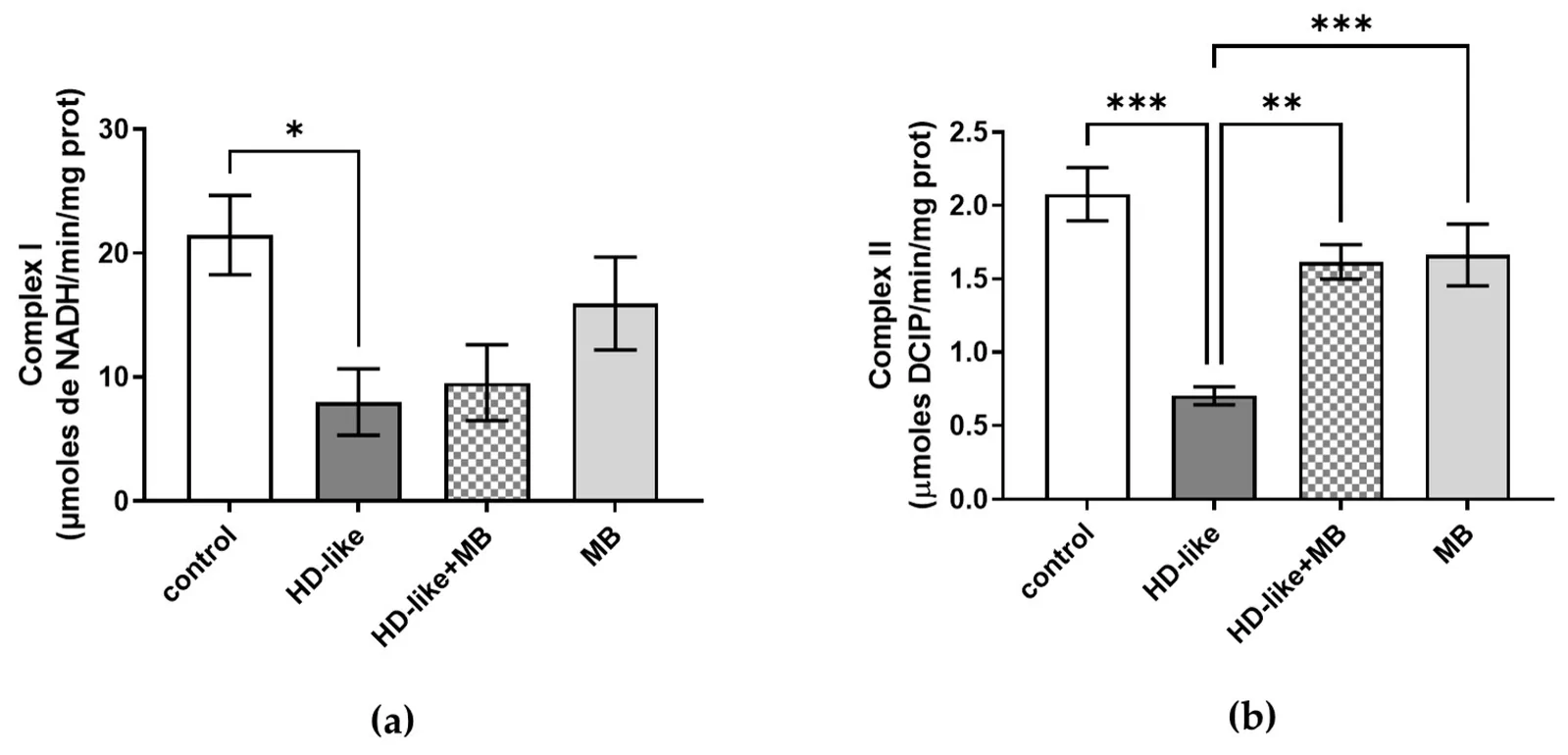
Effect of methylene blue on mitochondrial complex I and II activities in the striatum. (**a**) Complex I activity. (**b**) Complex II activity. Groups: control (*n* = 5), HD-like (3-NP, *n* = 6), HD-like + MB (3-NP plus methylene blue, *n* = 5), MB (methylene blue alone, *n* = 6). Data are presented as mean ± S.E.M. Statistical comparisons were performed using one-way ANOVA followed by Tukey’s post hoc test. * *p* < 0.05, ** *p* < 0.01, and *** *p* < 0.001 for the comparisons indicated.

To assess the effects of methylene blue (MB) on mitochondrial alterations associated with the HD-like condition, mitochondrial bioenergetic parameters were evaluated in isolated striatal mitochondria. Complex I activity was lower in the HD-like group than in the control group, approximately 60% (*p* = 0.03). No significant differences were observed between the HD-like + MB and HD-like groups (Figure 4a). In contrast, complex II activity was markedly reduced by 66% in the HD-like group relative to the control group (*p* < 0.0001). MB treatment in the HD-like group increased complex II activity by 43% compared to the untreated HD-like group (*p* = 0.001), reaching levels comparable to those in the control group (Figure 4b).

**Figure 5:**
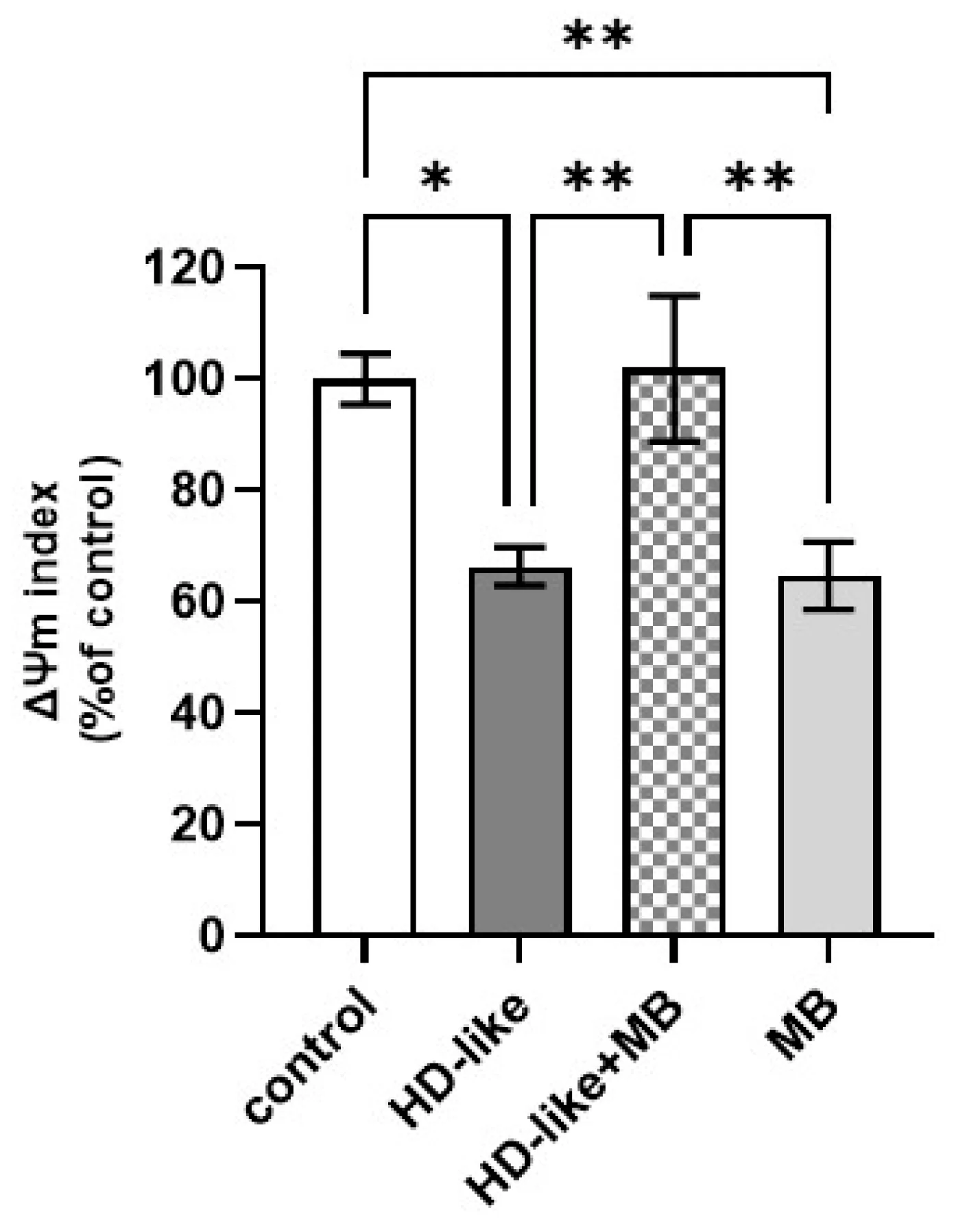
Striatal mitochondrial membrane potential index (ΔΨm index), assessed by CCCP-sensitive safranin quenching (%) following succinate addition. Groups: control (*n* = 5), HD-like (3-NP, *n* = 6), HD-like + MB (3-NP plus methylene blue, *n* = 5), MB (methylene blue alone, *n* = 6). Data are presented as mean ± S.E.M. Statistical comparisons were performed using one-way ANOVA followed by Tukey’s post hoc test. * *p* < 0.05 and ** *p* < 0.01 for the comparisons indicated.

Striatal mitochondrial membrane potential (Δψm) was monitored during succinate-driven, complex II-supported respiration. The striatal mitochondrial membrane potential index (Q) was significantly lower in HD-like animals than in controls (*p* = 0.0125; Figure 5). MB-treated HD-like animals showed a higher Q than untreated HD-like animals (*p* = 0.0076), whereas no significant difference was detected between the HD-like + MB and control groups (*p* = 0.9979). Animals receiving MB alone also exhibited a lower Q than controls (*p* = 0.0081).

**Figure 6:**
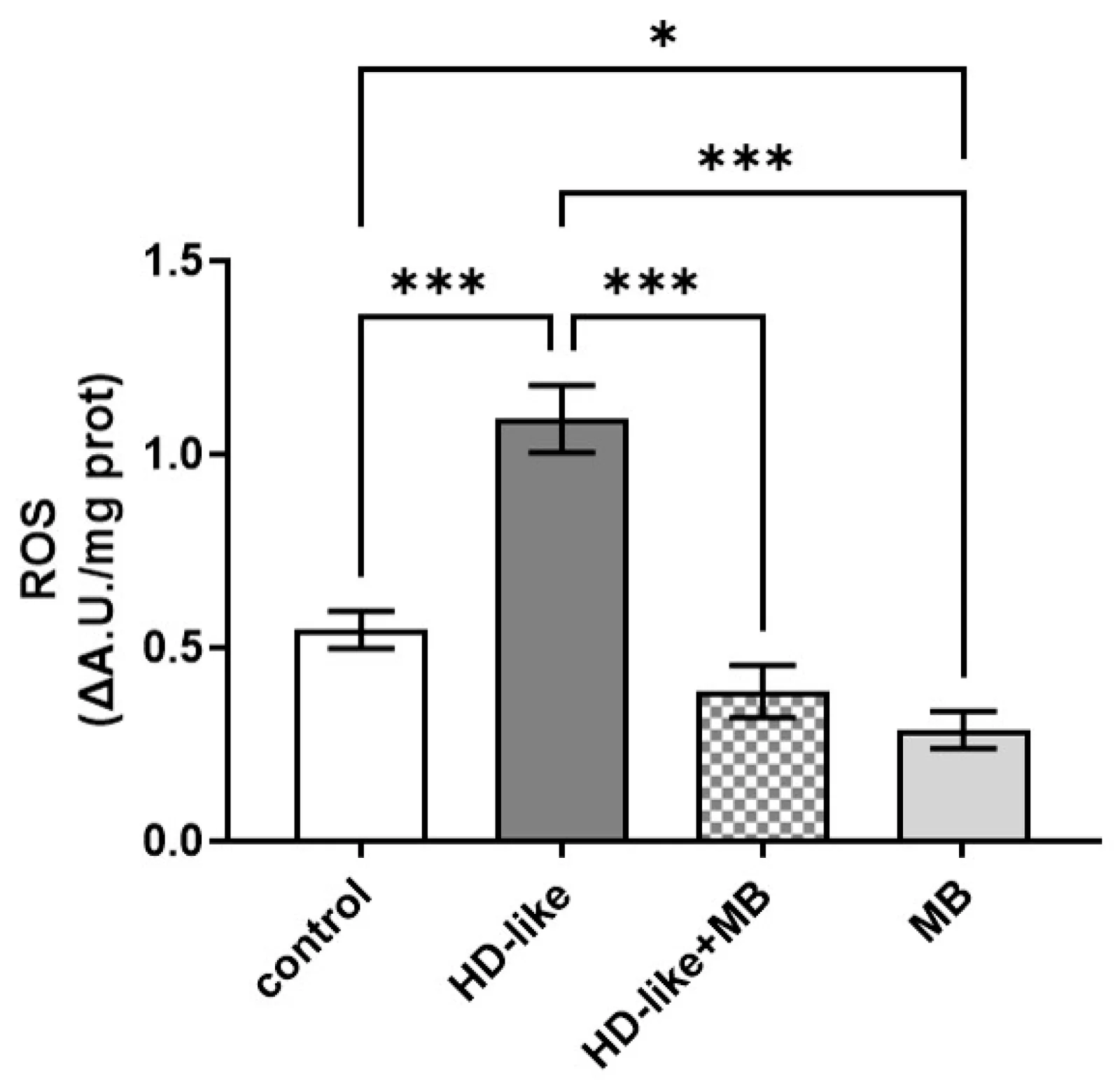
Striatal mitochondrial reactive oxygen species (ROS) production during succinate-supported respiration. A.U., arbitrary units. Groups: control (*n* = 5), HD-like (3-NP, *n* = 6), HD-like + MB (3-NP plus methylene blue, *n* = 5), MB (methylene blue alone, *n* = 6). Data are presented as mean ± S.E.M. Statistical comparisons were performed using one-way ANOVA followed by Tukey’s post hoc test. * *p* < 0.05 and *** *p* < 0.001 for the comparisons indicated.

Reactive oxygen species (ROS) production was quantified in striatal mitochondria during succinate-driven, complex II-supported respiration. The HD-like group exhibited approximately 100% higher ROS levels than the control group (*p* < 0.0001). MB treatment in HD-like + MB animals reduced ROS levels by 65% relative to the HD-like group (*p* < 0.0001), bringing them below control levels. The MB group also showed 48% lower ROS production than the control group (Figure 6).

### 3.3. Histological Analysis

**Figure 7:**
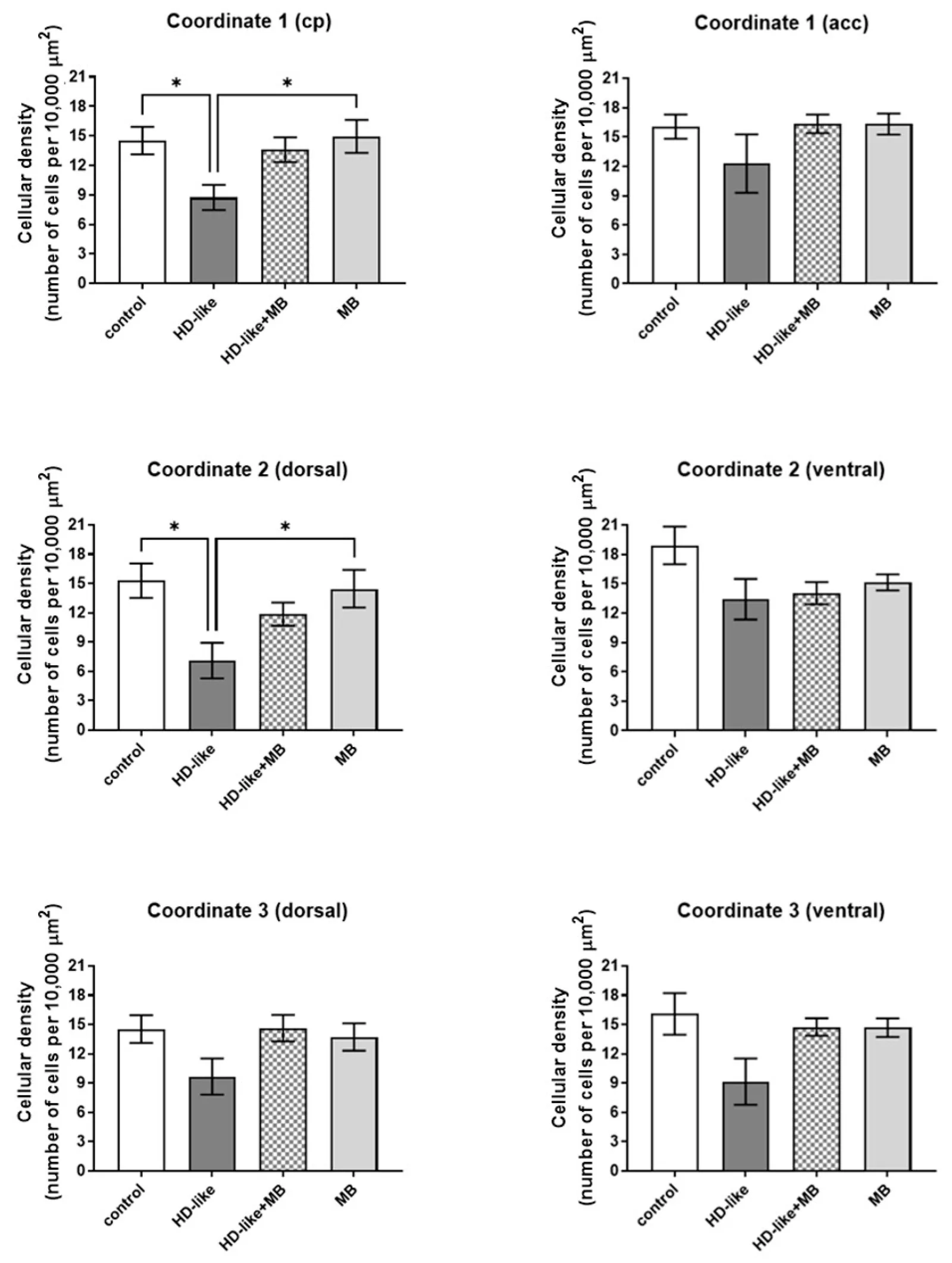
Effect of methylene blue on striatal nuclear density. Analyses were performed at the following stereotaxic coordinates: Coordinate 1, +1.70 mm from bregma; cp, caudate-putamen; acc, nucleus accumbens. Coordinate 2, +1.20 mm from bregma: striatum dorsal and ventral; and Coordinate 3, +0.70 mm from bregma, striatum dorsal and ventral. Groups: control (*n* = 5), HD-like (3-NP, *n* = 6), HD-like + MB (3-NP plus methylene blue, *n* = 6), MB (methylene blue alone, *n* = 5). Data are presented as mean ± S.E.M. Statistical comparisons were performed using one-way ANOVA followed by Tukey’s post hoc test. * *p* < 0.05 for the comparisons indicated.

**Figure 8:**
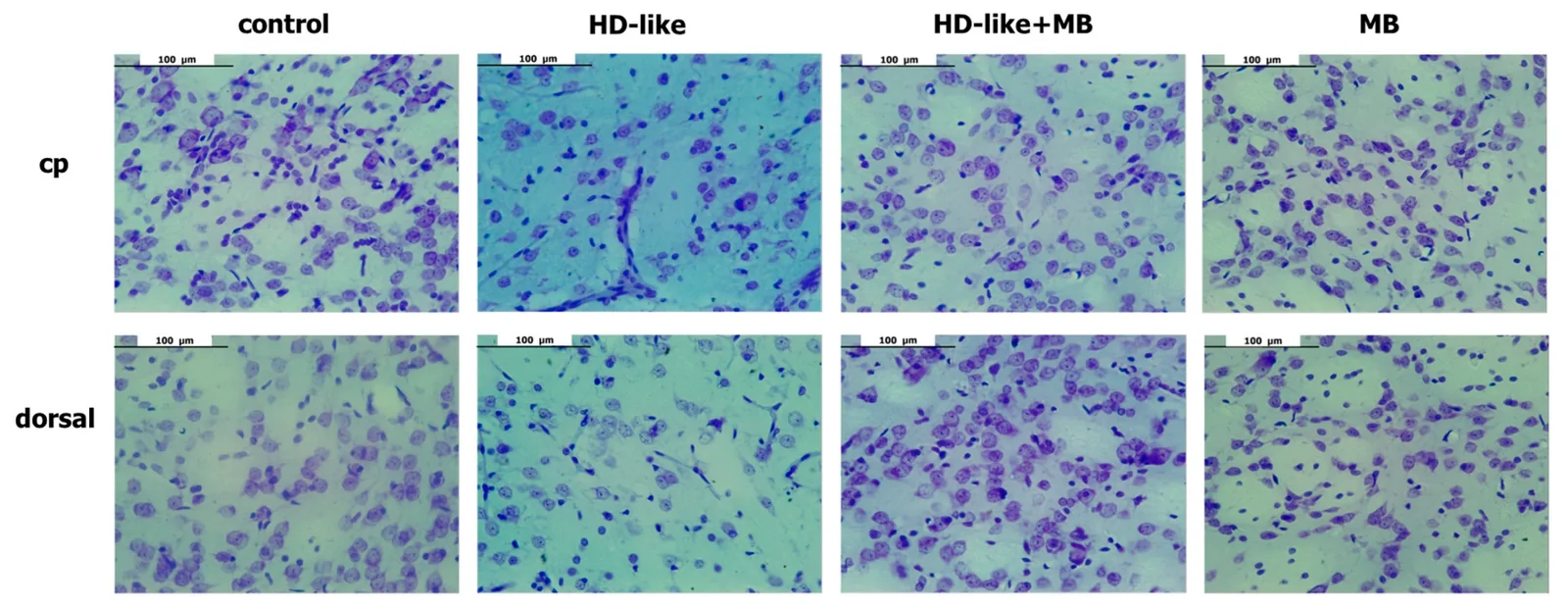
Representative cresyl violet-stained sections. Upper panels correspond to coordinate 1 in the caudate–putamen (cp), and lower panels to coordinate 2 in the dorsal striatum. Groups: control (*n* = 5), HD-like (3-NP, *n* = 6), HD-like + MB (3-NP plus methylene blue, *n* = 6), MB (methylene blue alone, *n* = 5). Scale bar = 100 µm.

Histological integrity of the striatum was evaluated by quantifying nuclear density. This approach enabled identification of structural changes associated with the HD-like condition and evaluation of methylene blue’s potential protective effects on tissue. Cresyl violet staining revealed a significant reduction in nuclear density in the caudate–putamen (cp) of the HD-like group compared with the control group (8.72 ± 1.28 vs. 14.52 ± 1.40, *p* = 0.026, coordinate 1), indicating cellular loss or cellular damage. Methylene blue treatment in the HD-like + MB group partially attenuated this effect, as nuclear density in the CP at coordinate 1 was significantly higher than in the HD-like group (13.59 ± 1.26 vs. 8.72 ± 1.28, *p* = 0.024). Differences were observed at coordinate 2 in the dorsal striatum, with lower nuclear density in the HD-like group compared with the control and MB groups (*p* = 0.023 and 0.031, respectively). However, MB treatment (HD-like + MB) did not improve this alteration. No significant differences were observed in the remaining regions analyzed (Figure 7 and Figure 8).

### 3.4. Motor Coordination, Balance, and Muscular Strength

**Figure 9:**
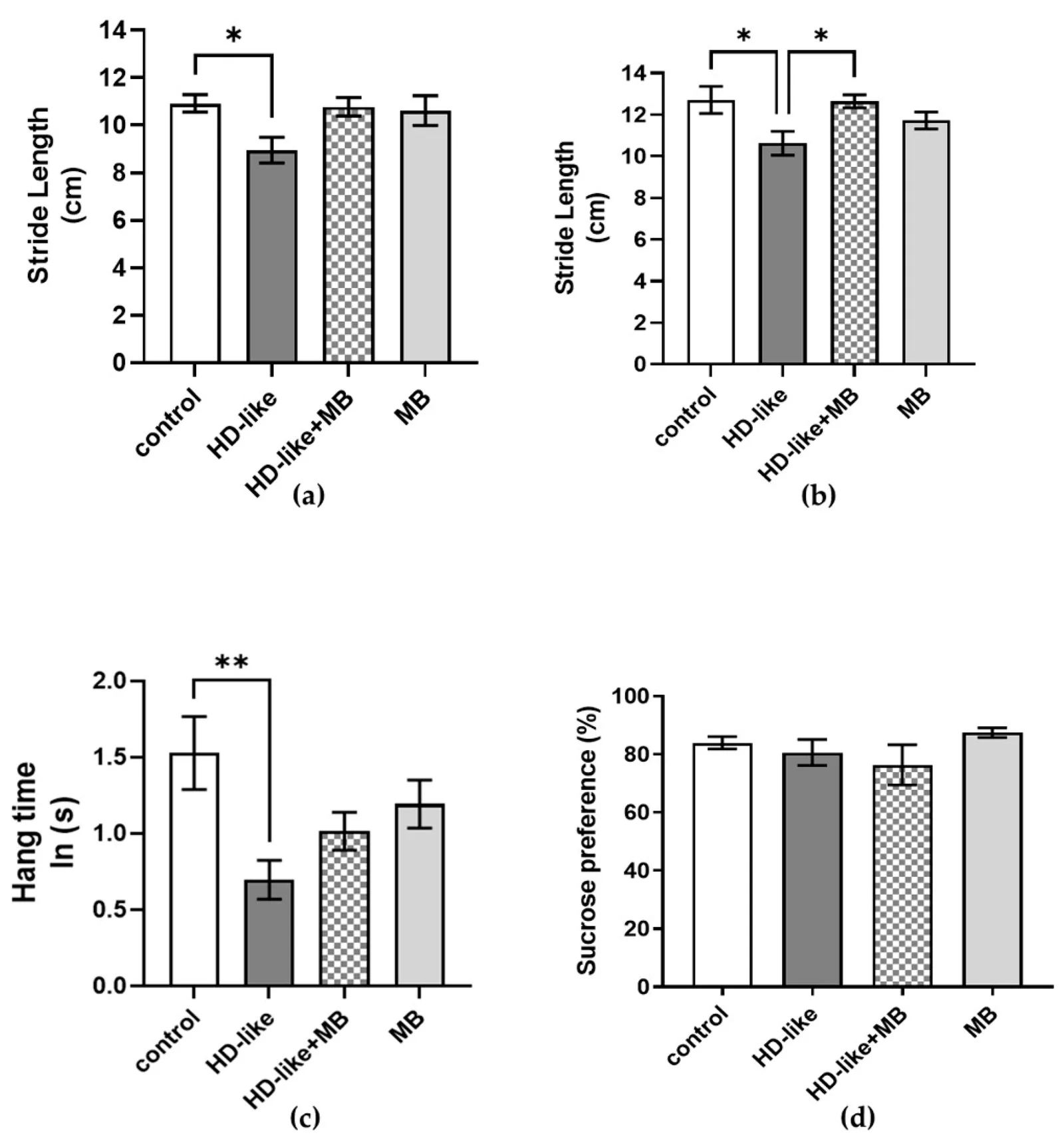
Effect of methylene blue on behavior. Gait performance assessed by the footprint test, (**a**) day 7 and (**b**) day 15. Performance in the horizontal grid test on day 15 (**c**) and sucrose preference (**d**). Groups: control (*n* = 14), HD-like (3-NP, *n* = 13), HD-like + MB (3-NP plus methylene blue, *n* = 13), MB (methylene blue alone, *n* = 16). Data are presented as mean ± S.E.M. Statistical comparisons were performed using one-way ANOVA followed by Tukey’s post hoc test. * *p* < 0.05 and ** *p* < 0.01 for the comparisons indicated.

Gait performance was assessed with the footprint test on days 7 and 15 of the experimental protocol. On day 7, the HD-like group showed significant alterations, with a shorter stride length than the control group (8.94 ± 0.5 vs. 10.92 cm; *p* = 0.041). Methylene blue treatment did not restore performance, as shown in Figure 9a (10.77 ± 0.3 cm; *p* = 0.058). On day 15, the HD-like group’s stride length remained significantly shorter than that of the control group (*p* = 0.028). Methylene blue treatment had a beneficial effect, as the HD-like + MB group showed significant improvement compared with the HD-like group (*p* = 0.042), indicating that the treatment ameliorated this behavioral impairment up to this time point (Figure 9b).

Motor coordination was assessed applying the rotarod test at day 18 of the experimental protocol. No significant differences were observed among groups at either time point or across any of the tested rotation speeds (the data for day 18 are shown in Figure S2).

Motor performance in the inclined plane test was evaluated on days 7 and 15. On day 7, the HD-like group showed impaired performance compared with the control group across all angles and orientations tested. We observed increased latency to climb up in the HD-like group compared with the control and MB rats (*p* ˂ 0.05). At the 15-day evaluation, we observed a minor latency to climb only at 45 degrees and in left-side orientation in the HD-like group compared with the control group and MB (*p* ˂ 0.05). Methylene blue treatment did not improve performance, no significant differences were observed between the HD-like and HD-like + MB groups across all tested conditions (Figures S3 and S4).

Performance in the horizontal grid test was assessed on days 7 and 15 of the experimental protocol. No significant differences were observed between groups on day 7. By day 15, the HD-like group showed impaired performance compared with the control group (0.69 ± 0.1 vs. 1.52 ± 0.2; *p* = 0.0057), as evidenced by reduced hang time. Methylene blue treatment did not improve performance, as no significant differences were observed between the HD-like and HD-like + MB groups at this time point (*p* = 0.054; Figure 9c).

**Figure 10:**
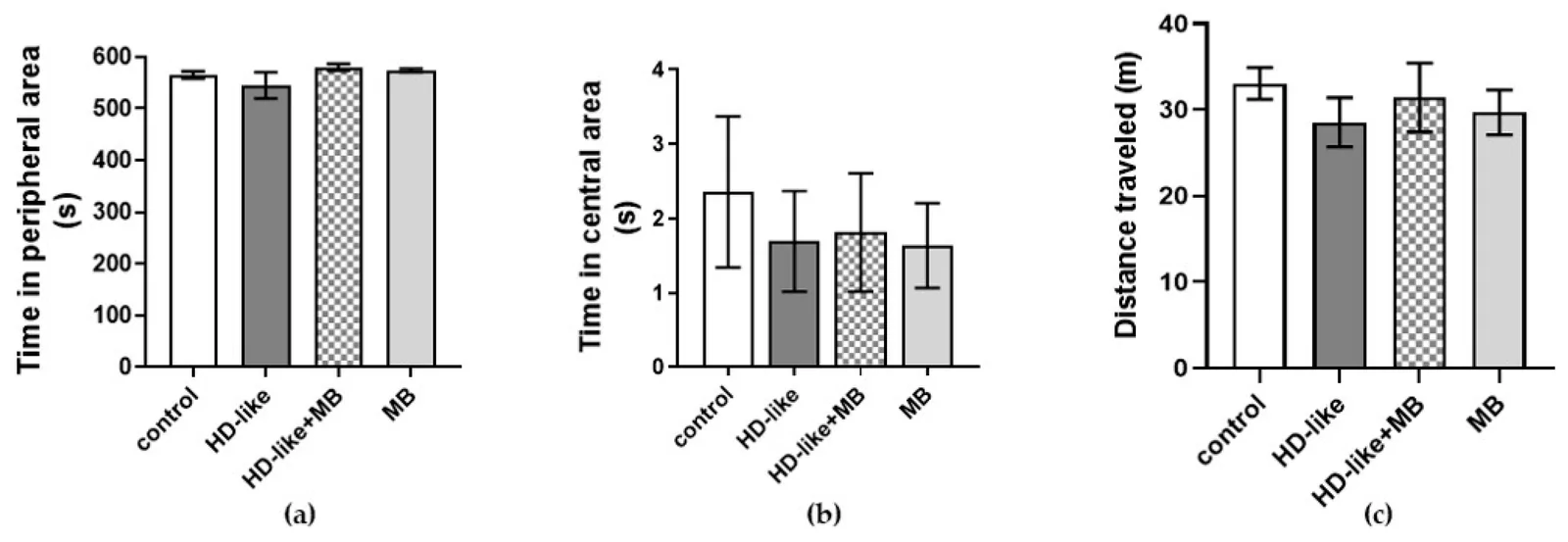
Effect of methylene blue on locomotor activity in the open field. Time in peripheral area (**a**), in the central area (**b**), and total distance traveled (**c**). Groups: control (*n* = 14), HD-like (3-NP, *n* = 13), HD-like + MB (3-NP plus methylene blue, *n* = 13), MB (methylene blue alone, *n* = 16). Data are presented as mean ± S.E.M. Statistical comparisons were performed using one-way ANOVA followed by Tukey’s post hoc test.

Spontaneous locomotor activity and exploratory behavior were evaluated in the open field by measuring the total distance traveled, as well as the time spent in the central and peripheral areas of the arena. No significant differences were observed among the experimental groups in any of the evaluated parameters (Figure 10), indicating that neither chronic 3-NP administration nor methylene blue treatment altered basal locomotor activity or exploratory behavior.

### 3.5. Anxiety-like Behavior, Anhedonia, and Spatial Working Memory

Sucrose preference was assessed to evaluate motivational state across experimental groups. No significant differences were observed among groups, as all groups exhibited comparable sucrose consumption, indicating similar hedonic behavior under the experimental conditions (Figure 9d).

**Figure 11:**
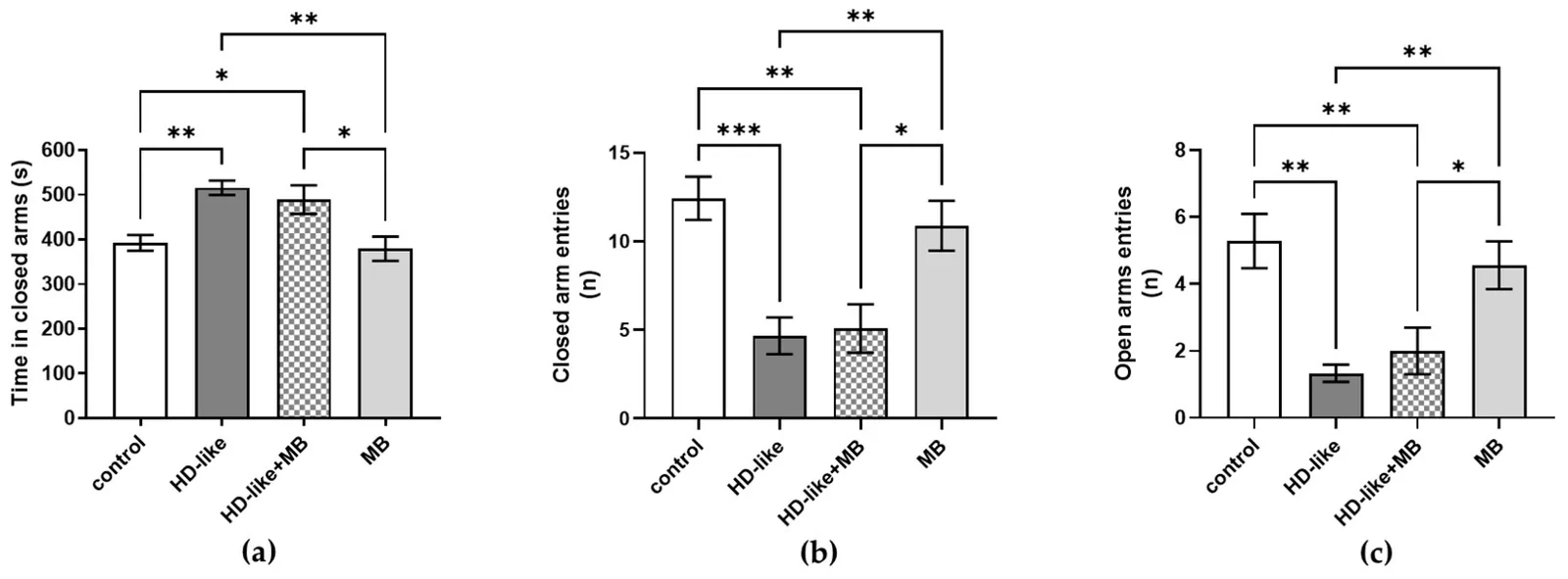
Effect of methylene blue on anxiety-like behavior in the plus maze. Time in closed arms (**a**), entries to closed arms (**b**), and entries to open arms (**c**). Groups: control (*n* = 14), HD-like (3-NP, *n* = 13), HD-like + MB (3-NP plus methylene blue, *n* = 13), MB (methylene blue alone, *n* = 16). Data are presented as mean ± S.E.M. Statistical comparisons were performed using one-way ANOVA followed by Tukey’s post hoc test. * *p* < 0.05, ** *p* < 0.01, and *** *p* < 0.001 for the comparisons indicated.

Anxiety-like behavior was assessed using the elevated plus maze by measuring time spent in the open and closed arms. The HD-like group spent significantly more time in the closed arms than the control group (*p* = 0.005), indicating increased anxiety-like behavior. Methylene blue treatment did not improve this parameter, as no significant difference was observed between the HD-like and HD-like + MB groups (*p* = 0.87). The control and MB groups exhibited similar behavior (*p* = 0.98). Furthermore, the number of entries into the closed and open arms was lower in the HD-like group than in the control and MB groups (Figure 11).

**Figure 12:**
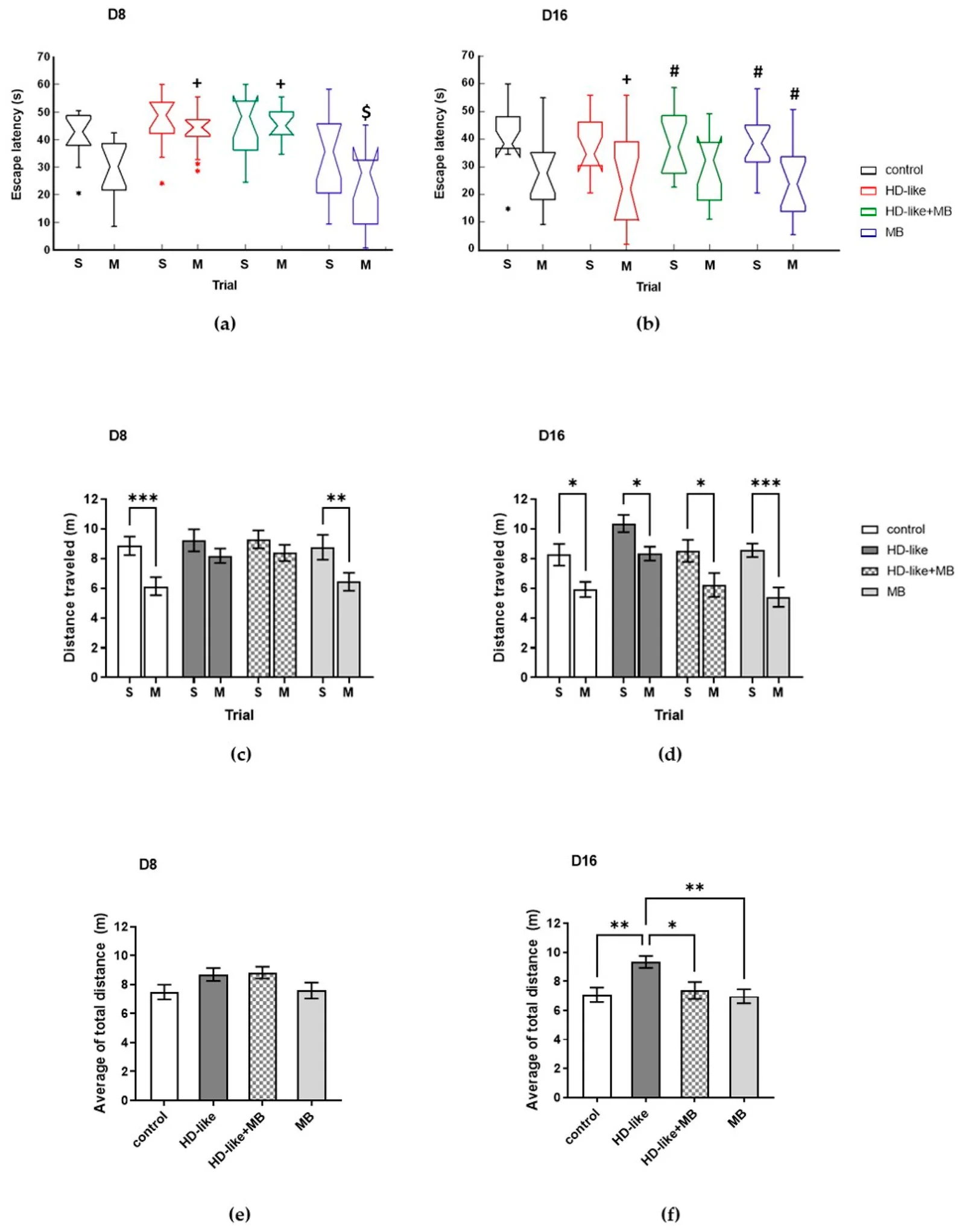
Effect of methylene blue on spatial working memory performance in the match-to-sample task. Escape latency was measured during sample (S) and match (M) trials on days 8 (**a**) and 16 (**b**). Groups: control (*n* = 14), HD-like (3-NP, *n* = 13), HD-like + MB (3-NP plus methylene blue, *n* = 13), MB (methylene blue alone, *n* = 16). (**a**) In the box-and-whisker plot, the lower boundary represents the 25th percentile, the horizontal line inside the box represents the median, and the upper boundary represents the 75th percentile. Statistical comparisons were performed using a Kruskal–Wallis test followed by Mann–Whitney U test for multiple comparisons. + different of control, # different of HD-like, and $ different of HD-like + MB for each trial. Distance traveled on days 8 (**c**) and 16 (**d**). Average total distance (sample + match) on days 8 (**e**) and 16 (**f**). Data are presented as mean ± S.E.M. Statistical comparisons used two-way ANOVA for repeated measures, followed by post hoc Bonferroni multiple comparisons ((**c**,**d**) data), and one-way ANOVA with Tukey’s post hoc test ((**e**,**f**) data). * *p* ˂ 0.05, ** *p* ˂ 0.01, *** *p* ˂ 0.001.

Performance on the spatial working memory task was assessed on day 8 of treatment. No significant differences were observed among groups in the escape latency during the sample trial. In the match trial, a significant group effect was detected (χ^2^ = 23.26, *p* < 0.0001). Post hoc analysis showed that both the HD-like and HD-like + MB groups had longer escape latencies than the control group (*p* = 0.0003 and *p* = 0.0001, respectively). Methylene blue treatment did not improve performance, as the HD-like and HD-like + MB groups showed no significant difference (*p* = 0.52). Moreover, a significant reduction in escape latency from sample to match trials was observed in the control (*p* = 0.0009) and MB (*p* = 0.0013) groups but not in the other two groups (Figure 12a). On day 16 of treatment, the HD-Like group had longer escape latencies than the control group (*p* = 0.0251) and the MB group (*p* = 0.0209) in the match group. However, in this evaluation, all groups showed reduced escape latencies, with more pronounced effects in the control (*p* = 0.0031) and MB (*p* = 0.0001) groups, followed by the HD-Like (*p* = 0.0034) and HD-like + MB (*p* = 0.0134) groups (Figure 12b).

Across trials (match vs. sample trial), total distance traveled on day 8 decreased consistently in the control and MB groups (*p* = 0.009 and *p* = 0.0026, respectively), and all groups’ performance improved on day 16 (control, *p* = 0.0112; HD-like, *p* = 0.0277; HD-like + MB, *p* = 0.0130; MB, *p* = 0.0001; Figure 12c,d), indicating that the MB-treated groups exhibited shorter travel distances.

Although no differences were observed on day 8 in the average of total distance (sample plus match trial), the HD-like group traveled longer distances on day 16 than the HD-like + MB (*p* = 0.0352), the control (*p* = 0.0099), and MB group (*p* = 0.0041), suggesting a protective effect of methylene blue on cognitive performance after 14 days of treatment (Figure 12e,f).

## 4. Discussion

The present results demonstrate that the early HD-like condition induced by 3-nitropropionic acid in low and chronic doses results in early mitochondrial dysfunction, oxidative stress, structural alterations, and behavioral impairments, with partial modulation observed at the mitochondrial and cognitive levels following methylene blue treatment. At the mitochondrial level, the reduction in complex I and II activities and loss of mitochondrial membrane potential, along with increased ROS production observed in the HD-like group, denote impaired electron transport and enhanced oxidative stress.

These findings support the presence of early bioenergetic dysfunction preceding motor and cognitive signs, and are consistent with previous reports of altered electron transport chain activity and increased oxidative stress in striatal neurons in early HD, both before motor symptoms and in the absence of motor symptoms [5,7].

The restoration of complex II activity and mitochondrial membrane potential, along with reduced ROS levels, following methylene blue treatment indicates improved mitochondrial electron transport efficiency and redox balance. Methylene blue acts as a redox cycler, accepting electrons from NADH-dependent sources and being reduced to leucomethylene blue (leucoMB), which can subsequently transfer electrons directly to downstream components such as cytochrome *c*. This alternative electron transfer supports electron flux and limits electron leakage, thereby maintaining mitochondrial respiration under conditions of electron transport chain dysfunction [20,34]. Experimental evidence has demonstrated that methylene blue can act as an alternative electron carrier capable of bypassing specific segments of the electron transport chain, reducing ROS and sustaining mitochondrial function [21,35]. Similar mechanisms have been described for other redox-active compounds, such as idebenone and short-chain quinones, which facilitate electron transfer through NQO1-dependent pathways under conditions of complex I dysfunction [36]. Despite these effects, methylene blue treatment did not restore complex I activity, indicating that its action does not involve direct recovery of enzymatic function as with idebenone and quinones. Instead, methylene blue supports mitochondrial respiration by facilitating alternative electron transfer to downstream components, thereby bypassing impaired segments of the electron transport chain without correcting the underlying enzymatic deficit. Notably, the extent of this bypass appears to be context-dependent, as some studies have reported that methylene blue does not fully restore respiration under conditions of complex III inhibition, suggesting that its efficacy depends on the site and severity of electron transport chain impairment [37]. In the present results, both complex II and mitochondrial membrane potential were restored by methylene blue treatment.

In addition, the reduction in mitochondrial ROS observed in the present study raises the possibility of involvement of redox-sensitive pathways such as Nrf2/ARE and AMPK, which regulate antioxidant responses and mitochondrial homeostasis. Consistent with this notion, methylene blue has been reported to modulate redox balance and activate Nrf2-dependent antioxidant pathways in neural models, thereby reducing oxidative stress and providing neuroprotection [38,39]. Further studies are required to determine whether Nrf2/ARE and AMPK pathways are directly engaged under these conditions.

Methylene blue restored mitochondrial membrane potential in the HD-like group, suggesting improved mitochondrial bioenergetics. Because mitochondrial membrane potential is essential for ATP production and cell survival, its preservation may indicate a more efficient electron transport chain. This effect aligns with methylene blue’s role as an alternative electron carrier, which improves electron flow and reduces oxidative stress. These findings support methylene blue’s potential as a mitochondrial-targeted neuroprotective agent during the prodromal stage of Huntington’s disease [20,34].

An interesting finding was the reduction in mitochondrial membrane potential in the MB group, despite the absence of other alterations. This effect may be related to methylene blue’s redox-cycling properties, which can alter electron flow and induce mild mitochondrial uncoupling, thereby reducing the membrane potential without compromising overall function. Under healthy conditions, this may represent a deviation from physiological balance, which explains why this was the only affected parameter. In the present study, ATP levels were not determined. Therefore, although the decrease in mitochondrial membrane potential did not coincide with alterations in the remaining mitochondrial parameters, it was not possible to establish whether this finding had any functional impact on mitochondrial energy production. Future studies including ATP quantification would help clarify the bioenergetic significance of this observation.

Histological analysis showed decreased nuclear density in the caudate–putamen, suggesting neuronal loss or damage in the HD-like group. This supports the known vulnerability of striatal medium spiny neurons, a characteristic feature of Huntington’s disease. According to neuropathological descriptions by Vonsattel and DiFiglia [40], HD-related neurodegeneration involves a progressive loss of striatal neurons, mainly in the caudate nucleus and putamen, along with gliosis and regional atrophy. Notably, these changes begin in specific striatal regions and worsen over time, indicating that structural damage starts early in the disease process. These findings are consistent with this, showing that histological changes are already detectable in the striatum at an early-like stage. Methylene blue treatment attenuated cellular loss in the caudate–putamen and provided partial protection in the dorsal striatum, where cellular density values were intermediate between those of the control and HD-like groups. This may reflect methylene blue’s ability to reduce early cellular stress without fully preventing damage to the striatum’s histological integrity.

Furthermore, in addition to histological alterations, the HD-like group exhibited reduced body weight, a feature commonly associated with disease progression and metabolic dysfunction in Huntington’s disease. Body weight loss has been linked to hypothalamic dysfunction, particularly the degeneration of orexin-positive neurons, which regulate food intake and the sleep–wake cycle. Moreover, alterations in Huntingtin-associated protein 1 (HAP1), a protein highly expressed in the hypothalamus and involved in intracellular trafficking of the epidermal growth factor receptor (EGFR), have been implicated in this process. Reduced HAP1 expression impairs EGFR signaling, contributing to hypothalamic neurodegeneration and subsequent weight loss [41,42]. Notably, methylene blue treatment at the dose employed did not stop the decrease in body weight, indicating that its impact on mitochondrial function was not strong enough to overcome these complex hypothalamic and metabolic changes. In this study, no systemic or peripheral markers related to energy metabolism or nutritional status were evaluated. Therefore, it was not possible to determine whether the observed body weight loss was exclusively associated with central hypothalamic alterations or whether peripheral metabolic changes also contributed to this phenotype.

Behavioral assessment further supported the presence of functional impairments associated with the early HD-like condition. Motor deficits were evident across multiple tests, reflecting impaired coordination and strength. Methylene blue treatment did not improve performance, suggesting that motor dysfunction may be less responsive to this intervention once established [43]. Interestingly, a distinct pattern emerged in gait performance. In the footprint test, methylene blue treatment improved motor coordination at later stages, indicating a selective effect on specific motor domains. This suggests that gait-related parameters may be more sensitive to early mitochondrial modulation than tasks requiring strength or complex motor integration. In the rotarod test, no significant differences were observed among groups at any time point or rotation speed evaluated, suggesting that motor coordination and balance were not sufficiently impaired under the conditions of this study. This lack of effect may reflect the rotarod’s limited sensitivity, which primarily detects deficits in advanced stages of motor dysfunction and may not capture subtle impairments present during early stages. In this context, it has been reported that early motor alterations in Huntington’s disease models are often task-dependent and may not be consistently detected by rotarod performance [43]. Additionally, a limitation of the present study is that the rotarod protocol used fixed rotation speeds in separate trials, rather than an automated accelerating paradigm (ramp protocol), which may be more sensitive for detecting subtle motor impairments. The lack of improvement with methylene blue treatment in this test further supports the idea that its neuroprotective effects are selective and may preferentially target cognitive and mitochondrial parameters rather than global motor coordination in the early stages.

In contrast, cognitive performance showed a more robust response to the MB. The HD-like group exhibited impairments on the spatial working memory task, indicated by increased escape latencies and distances. Importantly, methylene blue treatment improved cognitive performance, and the MB group consistently showed better outcomes. Notably, although an overall improvement was observed in all groups at day 16, this effect may be explained by task familiarization, as animals were previously exposed to the test on day 8. Therefore, improved performance at the later time point may reflect, at least in part, learning of the task. However, under these conditions, the HD-like + MB group performed better than the untreated HD-like group, suggesting that methylene blue enhances cognitive function beyond the effect of repeated exposure. In line with this, recent evidence supports the neuroprotective effect of methylene blue at low doses, including its role as a memory enhancer, its ability to increase BDNF production in animal models, its ability to reduce Huntingtin aggregation both in vitro and in vivo, and its ability to promote autophagy [19,20,21,44]. This supports the idea that cognitive processes may be more sensitive to early mitochondrial modulation, highlighting a potential therapeutic window during the early stage [45].

Regarding affective behavior, the HD-like group showed increased anxiety-like behavior in the elevated plus maze, whereas sucrose preference was not affected. Methylene blue did not reverse these parameters, suggesting that its effects may be domain-specific and insufficient to modulate emotional or motivational changes under these conditions. The absence of an effect on depressive and anxiety-like behavior may reflect the low dose of methylene blue used in this study (4 mg/kg), as higher doses (e.g., 15 mg/kg) have been reported to exert antidepressant-like effects comparable to those of imipramine, possibly through increased serotonin levels in limbic system regions [46]. Serotonergic signaling, particularly via 5-HT_1_A and 5-HT_2_ receptors, plays a central role in regulating anxiety-like behavior, and its dysregulation is strongly associated with affective disorders. In this context, methylene blue has been suggested to modulate monoaminergic systems, including serotonin pathways. MB inhibits MAO; however, these effects appear to be dose-dependent and may not be sufficient at low doses to produce anxiolytic outcomes. Interestingly, although higher doses appear more effective at improving affective behavior, they have not consistently been associated with cognitive enhancement, suggesting that the neurobehavioral effects of methylene blue are dose-dependent and may involve distinct underlying mechanisms. In contrast, the low dose used in the present study selectively improved cognitive performance without significantly affecting anxiety-like behavior [17,47,48].

Although the present findings support a selective neuroprotective effect of methylene blue at the mitochondrial and cognitive levels, their translational relevance remains limited by the current clinical evidence. Trials of MB-related compounds, particularly LMTM/HMTM, in Alzheimer’s disease and behavioral-variant frontotemporal dementia have yielded mixed results, with several studies failing to meet their prespecified primary clinical endpoints despite signals observed in selected subgroups, exposure-response, or biomarker analyses [22,23,24,25]. These findings are not directly comparable with those of the present study because the clinical programs mainly focused on pathological protein aggregation, whereas our work examines MB in a model of 3-NP-induced mitochondrial dysfunction. Even so, the clinical evidence indicates that the effects of methylthioninium compounds may vary with dose, formulation, disease context, and the mechanism being targeted.

Altogether, these findings support a model in which mitochondrial dysfunction precedes and contributes to structural damage and behavioral deficits emulating the early stage of the disease. The efficacy of methylene blue, particularly in cognitive performance, histological protection, specific aspects of motor coordination, and striatal mitochondrial function, suggests that targeting mitochondrial function may be a promising strategy in the early stages of the disease. However, its limited impact on body weight, global gross motor performance, and affective domains indicates that additional therapeutic approaches or combination strategies may be required to achieve broader neuroprotection.

The present results characterize the effects of MB under the experimental conditions used, although several questions remain. Additional doses should be evaluated to better define MB’s dose–response profile. Likewise, including an established pharmacological comparator in future experiments would help contextualize the magnitude of MB’s effects relative to other neuroprotective or mitochondrial-targeted interventions. Extending the treatment period may also help determine whether additional effects emerge with longer exposure. Finally, these results underscore the importance of early intervention, when mitochondrial-targeted therapies may be more effective at modifying disease progression.

## 5. Conclusions

The present study demonstrates that mitochondrial dysfunction, structural alterations, and behavioral impairments are evident during the early stage of the HD-like condition. Methylene blue treatment conferred neuroprotection, particularly at the mitochondrial, histological, and cognitive levels, supporting its potential as an early therapeutic strategy. However, its selective or differential effects on body weight, gross motor performance, and affective behavior highlight the disease’s complexity and suggest that additional or combined interventions may be required. Altogether, these findings underscore the importance of targeting early pathogenic mechanisms and reinforce the need for timely intervention to modify disease progression at early stages.

## Data Availability

The data presented in this study are available on request from the corresponding author due to restrictions (permissions must be authorized by the Mexican Institute of Social Security).

## Supplementary Materials

The following supporting information can be downloaded at: https://www.mdpi.com/article/10.3390/biology15201801/s1, Figure S1: Body weight changes over the treatment period. Data were analyzed using a two-way repeated-measures ANOVA with Tukey’s post hoc test. Groups: control (*n* = 14), HD-like (3-NP, *n* = 13), HD-like + MB (3-NP plus methylene blue, *n* = 13), MB (methylene blue alone, *n* = 16). Data are presented as mean ± S.E.M. Statistical comparisons were performed using one-way ANOVA followed by Tukey’s post hoc test. + HD-like different of control, * HD-like + MB different of control, *p* < 0.05, Figure S2: Effect of methylene blue on motor performance in the rotarod test. Groups: control (*n* = 14), HD-like (3-NP, *n* = 13), HD-like + MB (3-NP plus methylene blue, *n* = 13), MB (methylene blue alone, *n* = 16). Data are presented as mean ± S.E.M., Figure S3: Effect of methylene blue on motor performance in the inclined plane test at 30 and 35-degree angles. Groups: control (*n* = 14), HD-like (3-NP, *n* = 13), HD-like + MB (3-NP plus methylene blue, *n* = 13), MB (methylene blue alone, *n* = 16). Data are presented as mean ± S.E.M. Statistical comparisons were performed using one-way ANOVA followed by Tukey’s post hoc test. * *p* < 0.05, ** *p* < 0.01, and *** *p* < 0.001 for the comparisons indicated, Figure S4: Effect of methylene blue on motor performance in the inclined plane test at 40 and 45-degree angles. Control, HD-like (3-NP), HD-like + MB (3-NP plus methylene blue), MB (meth-ylene blue). Data are expressed as mean ± S.E.M. (*n* = 13–16 per group). One-way ANOVA with Tukey’s post hoc test, * *p* ˂ 0.05, ** *p* ˂ 0.01, *** *p* ˂ 0.001.
