# Supplementary material for: Early Administration of Methylene Blue Attenuates Mitochondrial Dysfunction and Cognitive Deficits in a Model of Huntington’s Disease
Source: Biology (Basel). 2026 Oct 9;15(20):1801. doi: 10.3390/biology15201801 (PMC13701637; doi:10.3390/biology15201801)
Supplement: Supplementary file 1 [file biology-15-01801-s001.zip › biology-4557170-supplementary.pdf]

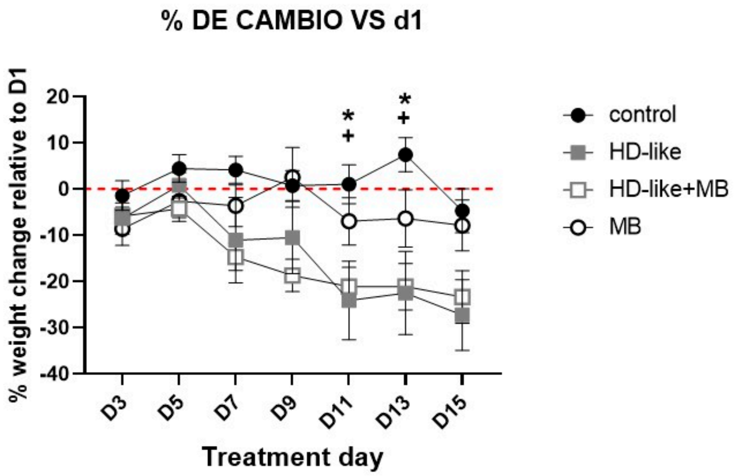

Figure S1. Body weight changes over the treatment period. Data were analyzed using a two-way repeated-measures ANOVA with Tukey's post hoc test. Groups: control (n= 14), HD-like (3-NP, n= 13), HD-like+MB (3-NP plus methylene blue, n= 13), MB (methylene blue alone, n= 16). Data are presented as mean ± S.E.M. Statistical comparisons were performed using one-way ANOVA followed by Tukey's post hoc test. + HD-like different of control, * HD-like+MB different of control, $p < 0.05$.

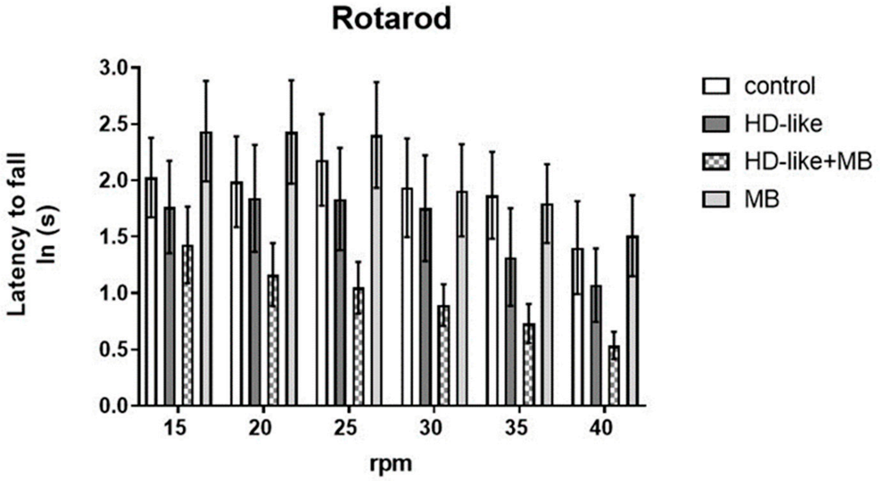

Figure S2. Effect of methylene blue on motor performance in the rotarod test. Groups: control (n= 14), HD-like (3-NP, n= 13), HD-like+MB (3-NP plus methylene blue, n= 13), MB (methylene blue alone, n= 16). Data are presented as mean ± S.E.M.

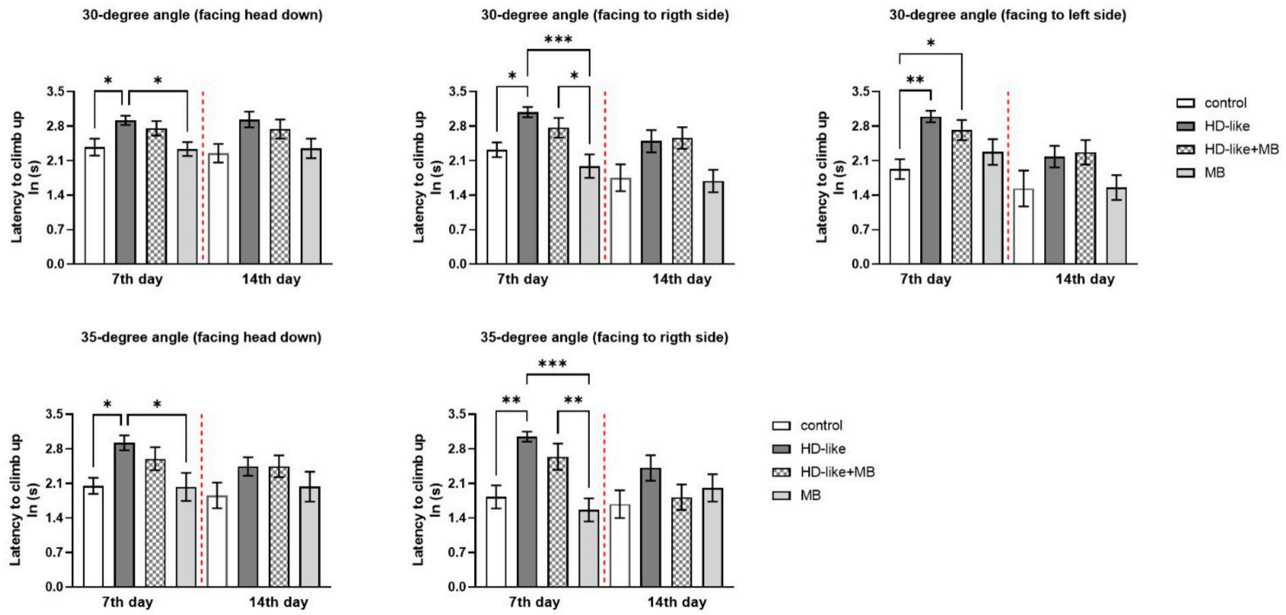

Figure S3. Effect of methylene blue on motor performance in the inclined plane test at 30 and 35-degree angles. Groups: control (n= 14), HD-like (3-NP, n= 13), HD-like+MB (3-NP plus methylene blue, n= 13), MB (methylene blue alone, n= 16). Data are presented as mean ± S.E.M. Statistical comparisons were performed using one-way ANOVA followed by Tukey's post hoc test. *p < 0.05, **p < 0.01, and ***p < 0.001 for the comparisons indicated.

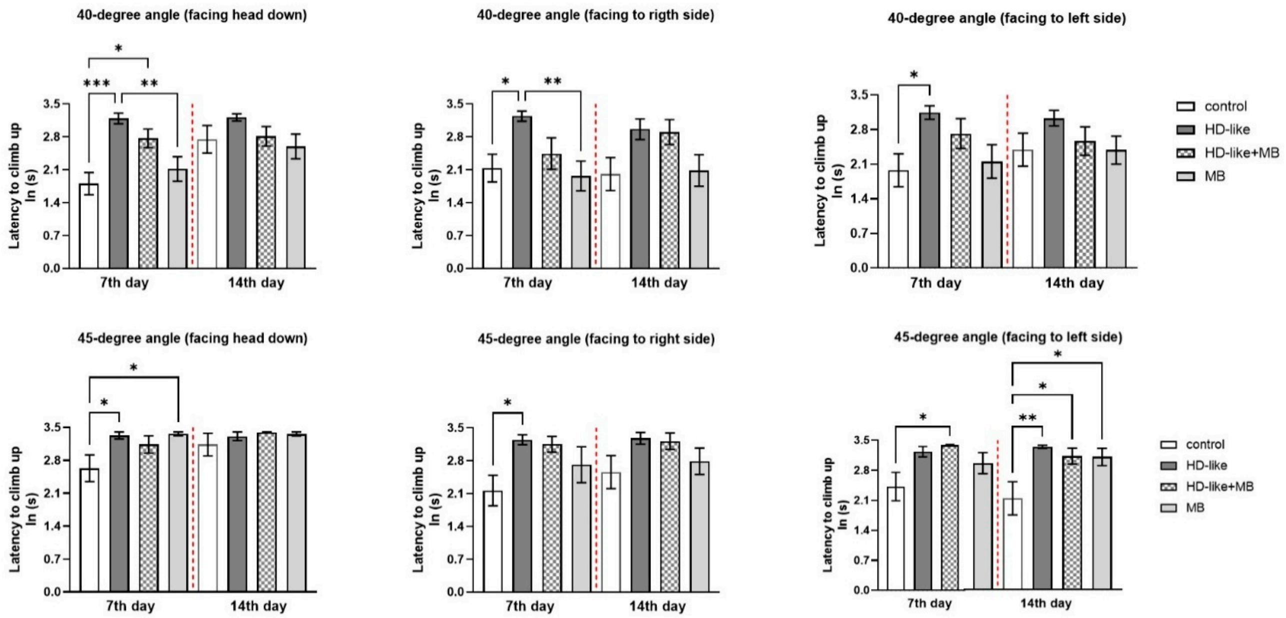

Figure S4. Effect of methylene blue on motor performance in the inclined plane test at 40 and 45-degree angles. Control, HD-like (3-NP), HD-like+MB (3-NP plus methylene blue), MB (meth-ylene blue). Data are expressed as mean ± S.E.M. (n = 13-16 per group). One-way ANOVA with Tukey's post hoc test, * p < 0.05, ** p < 0.01, *** p < 0.001.
